# Postpartum Loss to HIV Care and HIV Viral Suppression among Previously Diagnosed HIV-Infected Women with a Live Birth in New York State

**DOI:** 10.1371/journal.pone.0160775

**Published:** 2016-08-11

**Authors:** Carol-Ann Swain, Lou C. Smith, Denis Nash, Wendy P. Pulver, Victoria Lazariu, Bridget J. Anderson, Barbara L. Warren, Guthrie S. Birkhead, Louise-Anne McNutt

**Affiliations:** 1 AIDS Institute, New York State Department of Health, Albany, New York, United States of America; 2 School of Public Health, University at Albany, State University of New York, Albany, New York, United States of America; 3 School of Public Health, City University of New York, New York, New York, United States of America; 4 Center for Community Health, New York State Department of Health, Albany, New York, United States of America; 5 Institute for Health and the Environment, University at Albany, State University of New York, Albany, New York, United States of America; UCL Institute of Child Health, University College London, UNITED KINGDOM

## Abstract

Mother-to-child-transmission of HIV in the United States has been greatly reduced, with clear benefits for the child. However, little is known about factors that predict maternal loss to HIV care in the postpartum year. This retrospective cohort study included 980 HIV-positive women, diagnosed with HIV at least one year before pregnancy, who had a live birth during 2008–2010 in New York State. Women who did not meet the following criterion in the 12 months after the delivery-related hospital discharge were considered to be lost to HIV care: two or more laboratory tests (CD4 or HIV viral load), separated by at least 90 days. Adjusted relative risks (aRR) and 95% confidence intervals (CI) for predictors of postpartum loss to HIV care were identified with Poisson regression, solved using generalized estimating equations. Having an unsuppressed (>200 copies/mL) HIV viral load in the postpartum year was also evaluated. Overall, 24% of women were loss to HIV care during the postpartum year. Women with low participation in HIV care during preconception were more likely to be lost to HIV care during the postpartum year (aRR: 2.70; 95% CI: 2.09–3.49). In contrast, having a low birth weight infant was significantly associated with a decreased likelihood of loss to HIV care (aRR: 0.72; 95% CI: 0.53–0.98). While 75% of women were virally suppressed at the last viral load before delivery only 44% were continuously suppressed in the postpartum year; 12% had no viral load test reported in the postpartum year and 44% had at least one unsuppressed viral load test. Lack of engagement in preconception HIV-related health care predicts postpartum loss to HIV care for HIV-positive parturient women. Many women had poor viral control during the postpartum period, increasing the risk of disease progression and infectivity.

## Introduction

Public health campaigns in the United States (US) have effectively reduced mother-to-child-transmission (MTCT) of human immunodeficiency virus (HIV). In New York State (NYS), for example, only two cases of MTCT were reported in 2013 [1] compared to a peak of at least 475 cases in 1990. This remarkable reduction was achieved through vigorous efforts to identify HIV-positive women of childbearing age, including recommended HIV testing of all pregnant women followed by a second test during the third trimester, careful clinical management of HIV-positive mothers during pregnancy, rapid HIV testing during labor of pregnant women with no prenatal HIV test, screening of all newborns for exposure to HIV, and treatment for infants during the intrapartum and postpartum periods [2, 3]. While the health benefits to the child of avoiding perinatal HIV transmission are clear, less attention has focused on ensuring continuity of HIV medical care for the mother, particularly in the postpartum period.

Participation in HIV-related care among infected women appears to decline significantly after pregnancy [4]. The few US-based studies of retention in HIV-related care indicate that only 36% to 39% are engaged in appropriate HIV-related care during the postpartum year [5–7]. These previous studies provide important data on the prevalence of retention in care during the postpartum year. However, the studies were primarily urban based and their generalizability was limited to their local geography.

Global strategies call for a high level of retention in care and better virologic control among infected persons [8–10]. Reaching global benchmarks depends on understanding and addressing the needs of specific sub-groups, especially those who may experience major barriers to care and antiretroviral therapy (ART) adherence, such as postpartum women [11]. Previous studies have evaluated retention in HIV care, the primary objective of this study was to describe the complementary condition of being lost to HIV care (i.e., not retained in care). Our hypothesis was that engagement in HIV-related medical care in the preconception year and the timing of prenatal care initiation [5–7, 12] would be strong predictors of loss to HIV care in the 12 months after the delivery of a live infant. To quantify the overall health status of these women, we also measured the proportion with postpartum serum HIV RNA >200 copies/mL, a marker for increased transmission and adverse long-term health outcomes.

## Methods

### Study Population

A retrospective population-based cohort study was conducted to identify predictors of loss to HIV care in the postpartum year among previously diagnosed HIV-infected women with a live birth in NYS. Women were eligible for study inclusion if they: 1) delivered a live infant between 2008 and 2010; 2) were NYS residents at delivery with sufficient residence information to identify the census tract; 3) were diagnosed with HIV at least one year before the first pregnancy in the study period; and 4) could be identified in both the New York State HIV Surveillance System and the New York State Perinatal Database. Women with missing or invalid data on key variables (e.g., maternal discharge date) were excluded. Only the first delivery in the study period for each woman was retained for analysis.

### Data Sources

The NYS HIV Surveillance System receives all laboratory test results for individuals residing or receiving HIV-related care in NYS as mandated by public health law. Known gaps in reporting exist and are tied to reporting exemptions (e.g., testing performed within clinical trials). Name-based HIV reporting was implemented in 2000 [13, 14]. Reporting of all confirmed positive HIV antibody tests, HIV nucleic acid tests, viral loads, CD4 counts/percent (unless unrelated to HIV), and genotype nucleotide sequences began in 2005 [15], enabling longitudinal monitoring of HIV-related healthcare utilization among individuals with HIV infection. Reported CD4 and HIV viral load laboratory tests were used as proxies for HIV medical care encounters [16, 17]. Laboratory data for CD4 and HIV viral loads encompassed January 2007 through December 2011 to ensure equal observation periods (i.e., 12 months pre- and postpartum) for study subjects.

The NYS HIV Perinatal Database routinely receives data on infants exposed to HIV during gestation or at labor and delivery from the NYS Newborn Screening Program, a laboratory-based program that screens newborns for over 40 disorders and includes HIV antibody testing [18]. HIV exposure data from newborn screening is supplemented with demographic and clinical information abstracted from the prenatal care, labor and delivery, newborn, and pediatric medical records as well as laboratory diagnostic testing of the infant. For this analysis, HIV-infected pregnant women with a live birth identified from the state’s HIV Perinatal Database were linked to the statewide HIV Surveillance System using a unique NYS-assigned identifier found in both datasets.

Five-year estimates of residential poverty and education were obtained from the American Community Survey [19, 20] and were assigned to study participants based on census tract of residence at delivery.

### Outcome Measures

The primary outcome was loss to HIV care during the postpartum year. Women who did not meet the following criterion in the 12 months after the delivery-related hospital discharge were considered to be lost to HIV care: two or more laboratory tests (CD4 or HIV viral load), separated by at least 90 days [8]; all others were considered to be retained in HIV care. The 12 months after delivery (i.e., the postpartum year) was calculated as the maternal discharge date (plus one day) to 12 months after the maternal discharge date. Laboratory tests occurring between the birth event and the maternal discharge date were excluded, as the timing of the laboratory testing could not be reliably distinguished from HIV care during the delivery hospitalization and on-going care.

HIV viral suppression was the secondary endpoint and was defined as a quantitative viral load test ≤200 copies/mL. Time spent above an HIV viral load of 200 copies/mL was evaluated using methodology adapted from Gardner, et al. [21]. For women with two or more tests, the number of days between sequential pairs of tests was calculated, as was the range in viral load values between pairs. For instance, if the value of both tests in the pair was less than 200 copies, then the number of days spent above 200 copies equaled 0. If the value of both tests in the pair was 200 copies or greater, then the number days spent above 200 copies was equal to the number of days between pairs. For pairs with discordant HIV RNA values spanning 200 copies the number of days was calculated as:

$$\left(\frac{\text{higher viral load value}-200}{\text{higher}-\text{lower value}}\right)\text{*}(\text{difference }\left(\text{in days}\right)\;\text{between pairs}).$$

### Study Covariates

The factors studied include maternal demographic and clinical characteristics as well as infant health status (Tables 1 and 2). Infant data, maternal age at delivery, census tract residence at delivery and pregnancy-related variables were from the HIV Perinatal Database. The census tract at delivery was identified by geomatching the maternal address at delivery to a reference file containing census tract and latitude/longitude coordinates. All other study variables were from the statewide HIV Surveillance System.

**Table 1:** Socio-Demographic Characteristics and Lost to HIV Care in the Postpartum Year among HIV-Infected Women with a Live Birth, Diagnosed Before Pregnancy, New York State, 2008–2010.

		Lost to HIV Care^b^			
	Total^a^n = 980	Yes n = 232 (24%)	No n = 748 (76%)	Crude RR(95%CI)^c^	χ^2^ p-value^d^
	n (%)	n (%)	n (%)		
Residence at delivery					.27
NYS, excluding NYC	195 (20%)	52 (27%)	143 (73%)	1.16 (0.89–1.52)	
NYC	785 (80%)	180 (23%)	605 (77%)	Referent	
Race/Ethnicity					.06
Black	531 (54%)	128 (24%)	403 (76%)	Referent	
White	74 (8%)	25 (34%)	49 (66%)	1.40 (0.98–1.99)	
Hispanic	301 (31%)	59 (20%)	242 (80%)	0.81 (0.62–1.07)	
Other/Unknown	74 (8%)	20 (27%)	54 (73%)	1.12 (0.75–1.68)	
Maternal age at delivery (in years)					.12
<25	214 (22%)	49 (23%)	165 (77%)	1.21 (0.86–1.72)	
25–29	232 (24%)	61 (26%)	171 (74%)	1.39 (1.00–1.93)	
30–34	264 (27%)	71 (27%)	193 (73%)	1.42 (1.04–1.96)	
≥35	270 (28%)	51 (19%)	219 (81%)	Referent	
Percent below poverty^e^					.47
<10%	134 (14%)	38 (28%)	96 (72%)	Referent	
10-<20%	214 (22%)	51 (24%)	163 (76%)	0.84 (0.59–1.20)	
20-<30%	261 (27%)	65 (25%)	196 (75%)	0.88 (0.62–1.24)	
30-<40%	202 (21%)	45 (22%)	157 (78%)	0.79 (0.54–1.14)	
≥40%	168 (17%)	33 (20%)	135 (80%)	0.69 (0.46–1.04)	
Missing/Unknown	1 (--)	0 (--)	1 (--)		
Percent >25 years with Bachelor’s degree or greater^f^					.25
<15%	439 (45%)	93 (21%)	346 (79%)	Referent	
15%-<30%	385 (39%)	99 (26%)	286 (74%)	1.21 (0.95–1.56)	
≥30%	155 (16%)	40 (26%)	115 (74%)	1.22 (0.88–1.68)	
Missing/Unknown	1 (--)	0 (--)	1 (--)		
Low birth weight					.08
Yes	224 (23%)	43 (19%)	181 (81%)	0.77 (0.57–1.04)	
No	751 (77%)	187 (25%)	564 (75%)	Referent	
Unknown	5 (--)	2 (--)	3 (--)		
Infant HIV infection status					.29
Infected	11 (1%)	1 (9%)	10 (91%)	0.40 (0.06–2.62)	
Uninfected	914 (99%)	206 (23%)	708 (77%)	Referent	
Unknown	55 (--)	25 (--)	30 (--)		

^a^ column percent shown

^b^ row percent shown

^c^ RR = relative risk of being lost to follow-up (i.e., not retained) compared to referent; CI = confidence interval.

^d^ χ^2^ p-value calculated for non-missing data.

^e^ Percent of persons living below poverty in the census tract of mother’s residence at delivery.

^f^ Percent of persons >25 year of age with a bachelor’s degree or greater living in the census tract of the mother’s residence at delivery.

**Table 2:** Lost to HIV Care in the Postpartum Year among HIV-Infected Women with a Live Birth, Diagnosed Before Pregnancy, New York State, 2008–2010, Pregnancy-Related and HIV Characteristics.

		Lost to HIV Care^b^			
	Total^a^ n = 980	Yes n = 232 (24%)	No n = 748 (76%)	Crude RR (95%CI)^c^	χ^2^ p-value^d^
	n (%)	n (%)	n (%)		
Year of delivery					.97
2008	323 (33%)	78 (24%)	245 (76%)	1.01 (0.77–1.33)	
2009	356 (36%)	83 (23%)	273 (77%)	0.98 (0.74–1.29)	
2010	301 (31%)	71 (24%)	230 (76%)	Referent	
Trimester of first prenatal care visit					<.0001
First	439 (53%)	73 (17%)	366 (83%)	Referent	
Second	298 (36%)	79 (27%)	219 (73%)	1.59 (1.20–2.11)	
Third	64 (8%)	22 (34%)	42 (66%)	2.07 (1.39–3.08)	
No Care	24 (3%)	12 (50%)	12 (50%)	3.01 (1.91–4.72)	
Unknown	155 (--)	46 (--)	109 (--)		
Received ART during pregnancy					<.0001
Yes	940 (96%)	210 (22%)	730 (78%)	Referent	
No	37 (4%)	19 (51%)	18 (49%)	2.30 (1.64–3.21)	
Unknown	3 (--)	3 (--)	0 (--)		
Type of delivery					.45
Vaginal	383 (39%)	86 (22%)	297 (78%)	0.91 (0.72–1.15)	
C-section	594 (61%)	146 (25%)	448 (75%)	Referent	
Unknown	3 (--)	0 (--)	3 (--)		
Number of previous live births					.16
Zero	243 (29%)	45 (19%)	198 (81%)	Referent	
One	221 (27%)	57 (26%)	164 (74%)	1.39 (0.99–1.97)	
Two or more	362 (44%)	78 (22%)	284 (78%)	1.16 (0.84–1.62)	
Unknown	154 (--)	52 (--)	102 (--)		
Length of HIV diagnosis (in years)					.14
<2	20 (2%)	3 (15%)	17 (85%)	0.67 (0.24–1.93)	
2-<5	299 (31%)	82 (27%)	217 (73%)	1.23 (0.98–1.56)	
≥5	661 (67%)	147 (22%)	514 (78%)	Referent	
HIV transmission risk					.03†
Heterosexual	806 (82%)	203 (25%)	603 (75%)	Referent	
IDU	68 (7%)	14 (21%)	54 (79%)	0.82 (0.50–1.32)	
Perinatal infection	101 (10%)	14 (14%)	87 (86%)	0.55 (0.33–0.91)	
Other	5 (1%)	1 (20%)	4 (80%)	0.79 (0.14–4.60)	
Last CD4 before delivery					.49
0–350 cells/mm^3^	318 (33%)	76 (24%)	242 (76%)	1.09 (0.85–1.39)	
≥350 cells/mm^3^	639 (67%)	140 (22%)	499 (78%)	Referent	
Missing/Unknown	23 (--)	16 (--)	7 (--)		
Last HIV viral load before delivery					.02
≤200 copies/mL	690 (75%)	148 (21%)	542 (79%)	Referent	
>200 copies/mL	230 (25%)	66 (29%)	164 (71%)	1.34 (1.04–1.72)	
Missing/Unknown	60 (--)	18 (--)	42 (--)		
Median CD4 count (IQR)^e^	442 (290–607)	427 (275–604)	454 (296–607)	N/A	N/A
Median viral load (IQR)^e^	0 (0–200)	0 (0–380)	0 (0–151)	N/A	N/A
Lost to HIV care before (12 mos.) pregnancy					<.0001
Yes	685 (70%)	101 (15%)	584 (85%)	Referent	
No	295 (30%)	131 (44%)	164 (56%)	3.01 (2.42–3.76)	

^a^ column percent shown

^b^ row percent shown

^c^ RR = relative risk of being lost to follow-up (i.e., not retained) compared to referent; CI = confidence interval.

^d^ χ^2^ p-value calculated for non-missing data;

^†^χ^2^ p-value excludes persons in “Other” category.

^e^ Interquartile range, missing values excluded;

N/A = not applicable.

The 12 months prior to pregnancy was determined by estimating the date of conception (infant’s birthdate minus gestational age at delivery) and setting the observation period start date to 365 days prior. As with loss to HIV care after delivery, women who did not meet the following criterion in the 12 months before pregnancy were considered to be lost to HIV care before pregnancy: two or more laboratory test (CD4 or HIV viral load), separated by at least 90 days.

### Statistical Analysis

Ascential Quality Stage software was used to geomatch maternal addresses [22]. SAS V9.3 [23] and IVEware [24] were used to analyze the data. Bivariate analyses using χ^2^ tests were performed to investigate associations between categorical variables and lost to HIV care.

Imputation is the process of replacing missing data with plausible values from observed (i.e., non-missing) data. It is conducted to reduce biases associated with varying patterns of missing data that occur in research studies [25, 26]. Missing values were imputed for trimester of first prenatal care visit (16%), number of previous live births (16%), last viral load before delivery (6%), last CD4 before delivery (2%), type of delivery, low birth weight, percent below poverty, and percent aged 25 years and older with a bachelor’s degree or greater (<1%, respectively). Missing data was assumed to be missing at random [25, 26]. Multiple imputation of missing data [27] using Sequential Regression Multivariate Imputation [28] was implemented using IVEware.[24]. IVEware uses a posterior predictive distribution, which imputes missing values variable by variable incorporating data on all observed and imputed variables. Sequential imputation builds dependence between imputed values by using the correlation among study variables [28]. Five regression imputation cycles were performed for each variable imputed. Poisson regression [29, 30], solved using generalized estimating equations, was used to evaluate which study variables best predict lost to HIV care. Generalized estimating equations were used to adjust for dependencies among participants living in the same census tract [31]. Only variables with a significant p-value (<0.05) or those which improved model fit were retained.

A supplemental, bivariate analysis was conducted to compare NYS findings for to findings reported in the HIV literature. Similar to one previous investigation [7], receipt of HIV care was defined as at least one test in each six-month interval in the 12 months after delivery, separated by at least 60 days. Unlike the previous investigation which included women diagnosed less than two years prior to pregnancy, the supplemental analysis included only women diagnosed at least one year before pregnancy.

This study was approved by the NYS Department of Health Institutional Review Board. Informed consent was not required. The study used data reported to the NYS HIV Surveillance Registry; only census tract of residence at delivery and date of birth were retained in the analysis file, other identifying information was removed prior to analysis and there was no contact with infected persons.

## Results

In NYS, there were 1,546 HIV-infected women with a live birth between 2008 and 2010. Of these, 272 women were excluded due to a new HIV diagnosis during the first pregnancy in the study period or at delivery. Among the 92 women with a second delivery during the study period, only the first delivery was retained for analysis. Women who were diagnosed less than one year prior to conception (n = 75), had a missing maternal discharge date (n = 56), had inadequate address information (n = 33), could not be matched to the statewide HIV surveillance system (n = 26), did not reside in NYS (n = 7), or had other data errors (n = 5) were also excluded. The final study population was 980 HIV-positive women with a live birth.

Of the 33 women excluded from the analyses due to inadequate address information, 10 (30%) women were lost to HIV care in the postpartum year. The small number had little impact on the overall proportion of women who were lost to HIV care.

The study cohort was predominately Black (54%) and Hispanic (31%) and the median age at delivery was 30.5 years (interquartile range (IQR): 26–36 years) (Table 1). Most women resided in New York City (NYC) (80%) at delivery and two-thirds (65%) lived in census tracts where household poverty exceeded 20% of the federal poverty level. Nearly all (97%) women had at least one prenatal care visit during pregnancy. The majority began prenatal care in the first (53%) or second (36%) trimester (Table 2). Heterosexual contact (82%) was the primary risk factor for HIV transmission followed by perinatal infection (10%). The median time between HIV diagnosis and delivery was 7.3 years (IQR: 4.3–10.9 years) and 6.7 years (IQR: 4.0–9.5 years) when women who were themselves infected through perinatal contact were excluded. Seven women died in the first year after delivery, three within one month of delivery. There were cases of 11 mother-to-child transmission in this study.

Seventy-six percent (n = 748) of women were retained in care in the year after delivery. Among women who were lost to HIV care (n = 232), 82% had one (n = 83) or no (n = 107) laboratory result reported in the postpartum year; 18% (n = 42) had 2 or more tests separated by less than 90 days. In bivariate analyses, trimester of prenatal care initiation (second RR: 1.59; 95% CI 1.20–2.11, third RR: 2.07; 95% CI 1.39–3.08, no care RR: 3.01; 95% CI 1.91–4.72 compared to first trimester), no ART during pregnancy (RR: 2.30; 95% CI 1.64–3.21 compared to yes), last viral load before delivery (>200 copies/mL RR: 1.34; 95% CI 1.04–1.72 compared to ≤200 copies/mL), and lost to HIV care in the 12 months before pregnancy (yes RR: 3.01; 95% CI 2.42–3.76 compared to no) were significantly associated with a higher likelihood of being lost to HIV care in the postpartum year (Tables 1 and 2). Compared to women infected through heterosexual contact, women who were themselves infected through perinatal transmission were significantly less likely to be lost to HIV care in the postpartum year (RR: 0.55; 95% CI 0.33–0.91) in bivariate analysis. The statistically significant association between lost to HIV care before and after pregnancy remained after women who were infected through perinatal transmission were excluded (RR: 2.98; 95% CI 2.37–3.74).

After multivariable adjustment, women who were lost to HIV care in the year prior to pregnancy were significantly more likely to be lost to HIV care during the postpartum year (aRR: 2.70; 95% CI: 2.09–3.49) (Table 3). Women delivering a low birth weight infant were less likely to be lost to HIV care in the postpartum year (aRR: 0.72; 95% CI: 0.53–0.98).

**Table 3:** Multivariable Predictors of Lost to HIV Care in the Postpartum Year among HIV-Infected Women with a Live Birth, Diagnosed Before Pregnancy *.

	Adjusted RR**	95%CI	χ^2^ p-value
Lost to HIV care before (12 mos.) pregnancy			
Yes	Referent		
No	2.70	2.09 to 3.49	<.0001
Low birth weight			
Yes	0.72	0.53 to 0.98	.03
No	Referent		
Trimester of first prenatal care visit			
First	Referent		
Second	1.27	0.93 to 1.76	.10
Third	1.34	0.88 to 2.06	.14
No Care	1.73	0.95 to 3.17	.05

*Only significant variables or variables that improve model fit shown.

**adjusted RR = relative risk of loss to follow-up compared to referent, CI = confidence interval.

Variables considered in model selection include all variables listed in Tables 1 and 2, except ART during pregnancy and infant’s HIV infection status.

In the supplemental analysis, receipt of HIV care was defined as at least one test in each six-month interval in the 12 months after delivery, separated by at least 60 days. The results indicated that 72% and 73% of postpartum women met this criteria statewide, and in NYC, respectively.

Most women (75%) had a suppressed viral load at the last test before delivery but many did not sustain HIV viral suppression in the postpartum year. Among the 690 women who were suppressed at the last test before delivery 12% had no viral load test reported in the postpartum year and 44% had at least one unsuppressed HIV viral load test; only 44% were continuously suppressed. Thirty percent of women were unsuppressed by the first test in the postpartum year and the first test exceeded 1,000 copies/mL for one in five (22%) women. The majority of viral load tests occurred within the first (66%) or second (22%) quarter after discharge. Overall, 17% of women in the study population had no viral load tests in the postpartum year and 50% were unsuppressed on at least one test in the postpartum year; 33% were continuously suppressed

Time above 200 copies was calculated for women with at least two HIV viral load tests in the postpartum year (n = 671). The median observation time was 230 days (IQR: 161–273 days). There were 406 women (60%) with time above the 200 copies/mL threshold (Table 4); 40% had no time above 200 copies/mL indicating only suppressed viral load tests in the postpartum period. Among women with time above 200 copies/mL the median number of days above this threshold was 189.4 days (IQR: 122–253 days). Age, year of delivery and mode of HIV infection were significantly associated with time above 200 copies/mL (Table 4). Age and time above 200 copies/mL were inversely correlated. Even after excluding women with perinatal infection, younger women were more likely to be above this threshold as compared to women aged ≥35 years (p value: <0.0001).

**Table 4:** HIV Viral Suppression in the Postpartum Year among Women with ≥2 HIV Viral Load Tests.

	Viral Load Tests in Postpartum Year				
	Total n = 671	Any time above 200 copies/mL n = 406 (60%)	No time above 200 copies/mL n = 265 (40%)	Crude RR (95%CI)*	χ^2^ p-value
	n (%)	n (%)	n (%)		
Residence at delivery					.73
NYS, excluding NYC	131 (20%)	81 (62%)	50 (38%)	1.03 (0.88–1.20)	
NYC	540 (80%)	325 (60%)	215 (40%)	Referent	
Race/Ethnicity					.24
Black	349 (52%)	216 (62%)	133 (38%)	Referent	
White	48 (7%)	23 (48%)	25 (52%)	0.77 (0.57–1.05)	
Hispanic	227 (34%)	141 (62%)	86 (38%)	1.00 (0.88–1.14)	
Other/Unknown	47 (7%)	26 (55%)	21 (45%)	0.89 (0.68–1.17)	
Maternal age at delivery (in years)					<.0001
<25	146 (22%)	115 (79%)	31 (21%)	1.76 (1.47–2.10)	
25–29	150 (22%)	97 (65%)	53 (35%)	1.44 (1.19–1.75)	
30–34	181 (27%)	107 (59%)	74 (28%)	1.32 (1.08–1.61)	
≥35	194 (29%)	87 (45%)	107 (55%)	Referent	
Year of delivery					.0003
2008	229 (34%)	143 (62%)	86 (38%)	1.26 (1.06–1.50)	
2009	238 (35%)	162 (68%)	76 (32%)	1.37 (1.17–1.62)	
2010	204 (30%)	101 (50%)	103 (50%)	Referent	
Length of HIV diagnosis (in years)					.61
<2	14 (2%)	10 (91%)	4 (29%)	1.17 (0.84–1.65)	
2-<5	199 (30%)	117 (59%)	82 (41%)	0.97 (0.84–1.11)	
≥5	458 (68%)	279 (61%)	179 (39%)	Referent	
HIV transmission risk					.0002
Heterosexual	545 (81%)	310 (57%)	235 (43%)	Referent	
IDU	50 (7%)	34 (68%)	16 (32%)	1.20 (0.98–1.47)	
Perinatal infection	72 (11%)	60 (83%)	12 (17%)	1.47 (1.29–1.66)	
Other	4 (<1%)	2 (50%)	2 (50%)	0.88 (0.33–2.35)	

* RR = relative risk of having time above 200 copies/mL compared to referent, CI = confidence interval.

## Discussion

In our study, 24% of HIV-infected women with a live birth, diagnosed at least one year before conception, were lost to HIV care in the postpartum year; 76% were retained in care. The proportion retained approaches the 80% national goal [8]. The high prevalence of postpartum retention observed in NYS is likely due in part to the state’s public health program and policy infrastructure that effectively targets persons at high risk for HIV and HIV-infected women during pregnancy (e.g., HIV case management, outreach for early prenatal care and HIV risk reduction) [3, 32–34]. HIV prevention is integrated at multiple levels (i.e., individual, community and population), and across disciplines (medical care, prevention programs, and supportive services) [3]. For example, the Medicaid program provides comprehensive medical coverage for nearly half of all live births in NYS [35]; uninsured postpartum HIV-infected women receive services through safety net programs such as the HIV Uninsured Care Program [36]. The cumulative effects include a substantial reduction in new diagnoses and few mother-to-child transmissions each year [1, 37].

Three prior investigations have evaluated postpartum retention in care among HIV-infected US women [5–7]. All combined women diagnosed during pregnancy and women with long-standing HIV infection, precluding evaluation of factors that may contribute differently to postpartum engagement in care. In the present study, postpartum retention among HIV-infected women was nearly twice the prevalence reported by previous US investigations; only 1 in 5 were lost to HIV care. A recent population-based study of women who delivered in Philadelphia from 2005 to 2011 found that 39% were retained at one year [7]. Although attenuated, the NYS finding from the supplemental analysis was 72% retained statewide and 73% retained in NYC for 2008 to 2010. Viral suppression in the postpartum year could not be directly compared due to differences in methods between the two studies. The remaining two investigations were clinic-based and enrolled women in large urban areas in the Southeastern US. Clinic-based case ascertainment (versus the population-based cohort) may have contributed to some of the observed differences in the prevalence of retention in care as the clinic-based investigations did not track patient care outside of specific facilities.

Loss to HIV care in the 12 months before to pregnancy was the strongest predictor of suboptimal care postpartum. Programs that target women with this profile have the potential to achieve substantial reduction in lost to HIV care and improvement in clinical outcomes. For example, a recent study of persons at high risk for adverse HIV outcomes found significant improvement in retention in care and HIV viral suppression after a comprehensive HIV case management intervention [38]. The greatest improvement in that study was observed among individuals with no evidence of care in the time prior to enrollment. Pregnancy-related medical care encounters may be an optimal time to promote postpartum retention in HIV care. Interventions during these encounters could include a comprehensive assessment of HIV-related health care service use prior to pregnancy with particular focus on women with no care or suboptimal HIV-related care in the preconception year.

The majority of women (75%) were suppressed at the last test before delivery, one-fourth rebounded to >1,000 copies/mL by the first test postpartum, and half had at least one unsuppressed viral load in the postpartum year. This finding is consistent with previous studies which indicate that women may be more meticulous about healthcare and taking their ART during pregnancy than in the postpartum period [11, 39]. Although not evaluated in this study, factors such as mental health disorders, care giver responsibilities, and lack of economic and social support may also impede adherence to ART and contribute to subsequent poor virologic control [40, 41]. We also observed that age and poor viral control (viral load >200 copies/mL) were inversely correlated: a greater proportion of younger women had poor viral control compared to older women. This finding is comparable to previous work that shows a substantial proportion of infected persons have an unsuppressed viral load during the course of their infection with significant variation across age groups [42]. Women with time above the 200 copies/mL threshold are at higher risk for multiple deleterious outcomes, including viral transmission to uninfected partners [43]. Resources are needed to support durable virologic suppression among postpartum HIV-infected women.

Overall, most women had at least one prenatal care visit and initiated prenatal care in the first or second trimester of pregnancy. A fraction of these previously HIV-diagnosed women had no prenatal care or initiated care in the third trimester. This finding is comparable with national statistics which indicate that among the general population of pregnant women, few had no prenatal care or initiated care in the third trimester (6%) [44]. Consistent with previous studies [5, 6], we observed that lost to HIV care in the postpartum year increased significantly for women initiating prenatal care later in pregnancy. Previous work has also reported a higher likelihood of lost to HIV care among postpartum HIV-infected black women [6]. We found a higher percentage of white women were lost to HIV care in the postpartum year compared to women in other racial/ethnic groups. The reason for this finding was not apparent and needs further study. No association between sociodemographic variables and lost to HIV care was observed in multivariable analyses that included clinical factors.

Our data suggest one additional area of focus to minimize loss to HIV care. Almost 25% of women in the study delivered a low birth weight infant. These women were significantly less likely to be lost to HIV care in the postpartum year. Repeated infant-related healthcare encounters may be advantageous to maternal continuity of care, even if the encounters are tangential to the mother’s own care. Reduction in lost to HIV care in the postpartum year may be realized through interventions that target HIV-infected mothers during pediatric medical care encounters. In addition, co-location of maternal postpartum HIV medical care with general postpartum care and newborn care may further improve postpartum continuity of HIV care.

The following limitations should be considered when interpreting the study results. We relied on CD4 counts and viral load laboratory tests as proxies for HIV medical care. These proxy measures may over- or under- estimate the degree of retention in care [45]. In addition, emigration, care received in federal facilities, and care received from providers outside NYS are not fully captured by the NYS HIV Surveillance System and may also result in an overestimate of the true prevalence of lost to HIV care. Finally, the follow-up period was relatively short preventing long-term evaluation of HIV-related healthcare utilization.  

## Conclusions

This is one of the few US-based studies to assess loss to HIV care among HIV-infected women in the postpartum year at the population level. The study results show that there is substantial room for improvement in care coordination. Many of the women who were lost to HIV care in the postpartum year had at least one medical encounter during pregnancy. Women who had little to no evidence of HIV-related care in the year before pregnancy were most at risk for loss to HIV care during the postpartum year. In addition, many women had HIV viral loads above 200 copies/mL, increasing the risk of adverse long-term health outcomes and the likelihood of secondary transmissions of the virus. Thoughtful interventions are needed to address the complex needs of HIV-positive pregnant women to optimize treatment continuation during the postpartum year and thereafter. Proactive planning in advance of delivery to assess potential barriers to continuity of postpartum HIV primary care and coordination of care for this vulnerable population may further reduce both heterosexual transmission and poor health outcomes.
